# Shared genes between Alzheimer’s disease and ischemic stroke

**DOI:** 10.1111/cns.13117

**Published:** 2019-03-11

**Authors:** Chang‐Juan Wei, Pan Cui, He Li, Wen‐Jing Lang, Gui‐You Liu, Xiao‐Feng Ma

**Affiliations:** ^1^ Department of Neurology Tianjin Medical University General Hospital Tianjin China; ^2^ Tianjin Neurological Institute, Key Laboratory of Post‐neurotrauma Neuro‐repair and Regeneration in Central Nervous System Ministry of Education Tianjin China; ^3^ School of Life Science and Technology Harbin Institute of Technology Harbin China

**Keywords:** Alzheimer’s disease, gene expression analysis, gene‐based analysis, genome‐wide association studies, ischemic stroke, pleiotropic genes, Versatile Gene‐based Association Study‐2 version 2

## Abstract

**Aims:**

Although converging evidence from experimental and epidemiological studies indicates Alzheimer's disease (AD) and ischemic stroke (IS) are related, the genetic basis underlying their links is less well characterized. Traditional SNP‐based genome‐wide association studies (GWAS) have failed to uncover shared susceptibility variants of AD and IS. Therefore, this study was designed to investigate whether pleiotropic genes existed between AD and IS to account for their phenotypic association, although this was not reported in previous studies.

**Methods:**

Taking advantage of large‐scale GWAS summary statistics of AD (17,008 AD cases and 37,154 controls) and IS (10,307 IS cases and 19,326 controls), we performed gene‐based analysis implemented in VEGAS2 and Fisher's meta‐analysis of the set of overlapped genes of nominal significance in both diseases. Subsequently, gene expression analysis in AD‐ or IS‐associated expression datasets was conducted to explore the transcriptional alterations of pleiotropic genes identified.

**Results:**

16 AD‐IS pleiotropic genes surpassed the cutoff for Bonferroni‐corrected significance. Notably, *MS4A4A* and *TREM2*, two established AD‐susceptibility genes showed remarkable alterations in the spleens and brains afflicted by IS, respectively. Among the prioritized genes identified by virtue of literature‐based knowledge, most are immune‐relevant genes (*EPHA1*, *MS4A4A*, *UBE2L3* and *TREM2*), implicating crucial roles of the immune system in the pathogenesis of AD and IS.

**Conclusions:**

The observation that AD and IS had shared disease‐associated genes offered mechanistic insights into their common pathogenesis, predominantly involving the immune system. More importantly, our findings have important implications for future research directions, which are encouraged to verify the involvement of these candidates in AD and IS and interpret the exact molecular mechanisms of action.

## 1 INTRODUCTION

Alzheimer's disease (AD) is the world's leading cause of dementia. The hallmarks of AD are extracellular amyloid‐β peptide (Aβ) accumulation and intracellular neurofibrillary tangles (NFTs), the latter of which is formed by hyperphosphorylated tau protein. Along with the notorious reputation as the second leading cause of mortality and disability worldwide,^1^, ^2^ stroke is another major cause of age‐related cognitive decline and dementia.^3^ As the most prevalent form, ischemic stroke (IS) accounts for ~85% of stroke incidents.^3^ Collectively, AD and IS both exert a large burden on global public healthcare and clinical practice.

Growing evidence indicates that there are links between AD and IS. Firstly, emerging epidemiologic research shows that AD is associated with considerable increased risk of IS,^4^, ^5^ and vice versa.^6^ Secondly, neuropathological studies show that cerebrovascular lesions frequently coexist with AD pathology.^7^ The two mixed pathologies act synergistically in increasing the odds of clinical dementia.^8^ Indeed, a handful of studies have reported that brain ischemia is a non‐neglectable factor driving the development of AD through dysregulated expression of AD‐associated genes, such as Aβ precursor processing genes and tau protein gene.^9^, ^10^ Lastly, tau protein, a core hallmark of AD, can exacerbate brain injury in experimental IS through tau‐mediated iron export and excitotoxicity.^11^, ^12^ Taken together, we hypothesized that there might be a shared genetic basis underlying these connections between AD and IS.

Genome‐wide association studies (GWAS) have yielded new insights into the genetics of AD^13^, ^14^ and IS.^15^, ^16^ Shared genetic variants between AD and IS or its subtypes, have been first determined by Traylor et al.^17^ They tested whether established genome‐wide single nucleotide polymorphisms (SNPs) for AD or IS were significantly associated with the other disease. Yet no such variants have been found. Conventional GWAS methods just focus on significant SNPs judging by overly stringent criterion (*P* < 5.00E−08) when exploring the genome. There is an emerging consensus that, however, complex diseases are mostly driven by the joint action of a large proportion of SNPs having modest effects well below genome‐wide significance.^18^ Alternatively, gene‐based analysis can obtain more validated associations by combining the effects of all SNPs within corresponding genes, thus expand knowledge about genetic architectures of complex diseases.

Hence, in our present study, we performed gene‐based association tests to identify potential candidate genes shared between AD and IS. Next, gene expression analyses were conducted to evaluate shared genes’ expression alterations in AD and IS brains or peripheral blood versus matched controls. Furthermore, to further interpret the molecular mechanisms that underpin AD and IS, systematic dissection of individual genes’ functionalities was conducted.

## 2 MATERIALS AND METHODS

### 2.1 Samples

The GWAS statistics data for AD and IS analyses were from the International Genomics of Alzheimer's Project (IGAP)^13^ and the METASTROKE consortium of the International Stroke Genetics Consortium,^15^ respectively. Both GWAS datasets are based on populations of European descent imputed to the 1000 Genomes Project (1000G) reference panel. Poorly genotyped or imputed SNPs and SNPs with a minor allele frequency (MAF) of less than 0.01 were filtered out in both datasets.

IGAP is a large‐scale, two‐stage GWAS (for more details, see the research by Lambert et al^13^). In stage 1, a meta‐analysis of four published GWAS samples comprising 17,008 AD cases and 37,154 controls was conducted. After quality control, 7,055,881 SNPs were available for analysis. In stage 2, 11,632 SNPs showing moderate evidence of association (*P* < 1.00E−03) in stage 1 were genotyped and tested for replication in an independent sample totaling 8,572 cases and 11,312 controls. Lastly, a meta‐analysis combining results from stages 1 and 2 was performed. For the present study, we used only summary data from stage 1.

The METASTROKE collaboration genotyped and imputed approximately 9 million SNPs from a meta‐analysis of 12 independent GWAS comprising 10,307 IS cases and 19,326 controls. Differing from prior IS GWAS data imputed to the HapMap panel, which comprised up to 2.5 million SNPs with MAF more than 0.05, this expanded set of SNPs informed by 1000G project also included low‐frequency variants (MAF 0.01‐0.05) and totaled 8.3 million quality‐controlled SNPs for analysis. For more detailed information, refer to the original study.^15^

### 2.2 Statistical analyses

#### 2.2.1 VEGAS2 method

Using the GWAS summary data for AD and IS, we performed a gene‐based association test implemented in an updated version of Versatile Gene‐based Association Study‐2 version 2 (VEGAS2).^19^ Among various methods of gene‐based analysis, VEGAS2 is particularly feasible for analyzing GWAS summary statistics where individual‐level genotypic and phenotypic data are unavailable. By uploading the individual SNPs’ IDs and their association *P*‐values, VEGAS2 sums the effects of all the SNPs within a gene and corrects for linkage disequilibrium (LD) referring to 1000G reference set and thus, generates a gene‐based test statistic by doing simulations from the multivariate normal distribution. The simulation approach is computationally more efficient than other methods that rely on permutations, such as PLINK, minSNP.^20^ The default “symmetric boundaries ±0 kb outside gene and SNPs in LD above *r* ^2^ = 0.8” was chosen to define gene boundaries, which meant that the effects of SNPs within a gene, also outside of the gene with *r* ^2^ > 0.8 with the ones in the gene, were considered to calculate gene‐based *P*‐values. This option both took account into the effects of nearby regulatory SNPs and reduced the non‐specificity caused by large boundaries like ±50 kb.

To reduce the possibility of a single disease driving the cross‐disease associations and uncover truly pleiotropic genes shared by AD and IS, we focused on shared genes that were nominally significant in each disease (*P*_AD_ < 0.05 and *P*_IS_ < 0.05).

#### 2.2.2 Meta‐analysis using Fisher's method

We used Fisher's method to combine the *P*‐values calculated by the VEGAS2 for each gene shared by AD and IS. For a given gene, the Fisher formula for meta‐analysis is:

$$<![CDATA\left[x^{2}=-2\sum_{i=1}^{k}\ln\left(P_{i}\right)\right]]>$$

where *P_i_* are the *P*‐values of the genes in the *i*_th_ study and *k* is the total number of studies. *x* ^2^ follows a chi‐square distribution with 2k degrees of freedom.^21^ The gene‐based meta‐analysis was carried out using the R software package.

To avoid false positive signals, we applied the stringent Bonferroni correction accounting for the number of genes and phenotypes tested, that is, the significance threshold was set at 0.05/2N, where N represented the number of shared genes with nominal significance in both AD and IS.

#### 2.2.3 Gene expression analysis method

To explore the expression alterations of shared genes in each disease, we surveyed the expression datasets of AD and IS from the Gene Expression Omnibus (GEO) repository. Gene expression analysis was mainly restricted to brains and peripheral blood, as they are the most affected by AD‐ or IS‐associated pathology.

Because AD‐associated neuropathology shows regional specificity, expression profiles from discrete brain regions are more informative for discerning AD molecular signatures than analyses based on whole‐brain expression data. Thus, we examined expression data from separate regions from postmortem human AD and control brains, including the dorsolateral prefrontal cortex (PFC) and hippocampus regions. The former (GSE44770) sampled 549 brains of 376 late‐onset AD patients and 173 controls.^22^ The latter (GSE48350) comprised 19 AD cases versus 43 matched controls.^23^

For IS, samples of brains and other tissues (eg spleen) from patients were generally not available. Considering that the core features of IS pathophysiology in rodents and humans are analogous, we included the expression data from peri‐infarct brain areas of rats (GSE55260).^24^ We also analyzed transcriptional data from peripheral blood collected within 24 hours of stroke onset in 39 IS patients and 24 controls (GSE16561).^25^ Additionally, we explored transcriptional profiles from mouse spleens (GSE70841) as spleen is the major lymphoid organ involved in the inflammatory milieu secondary to brain ischemia. All gene aliases in rat or mouse were transformed to the official symbols corresponding to human genes.

If there were multiple transcripts within the same gene, the one with the smallest *P*‐value was selected. Due to between‐study heterogeneity, not all transcripts of AD‐IS pleiotropic genes appeared in each dataset. The differential expression was determined by Bonferroni correction accounting for the number of shared genes present in each dataset (*P* = 0.05/n, n ≤ 16).

## 3 RESULTS

### 3.1 Gene‐based testing for risk genes of AD and IS

Firstly, the VEGAS2 method was applied to individual GWAS summary data from AD and IS. For AD, 34 genes exceeded gene‐wide significance (*P* < 2.35E−6 for 21,244 gene tests). Apart from 20 genes at 19q13.31‐q13.32 harboring the well‐known *APOE* locus, we confirmed 12 genes (*CD33*, *ABCA7*, *HLA‐DRB6*, *MS4A2/MS4A6A*, *EPHA1*, *PICALM*, *CR1*, *CLU*, *MTCH2*/*SLC39A13*, and *BIN1*) within 10 established risk loci (*CD33*, *ABCA7*, *HLA‐DRB5‐DRB1* region, *MS4A *locus, *EPHA1*, *PICALM*, *CR1*, *CLU*, *CELF1*, and *BIN1*).^13^ Considering the other 39 genes outside the *APOE* locus passing a loose significance threshold (*P* < 1.00E−4), 23 genes belonged to the established risk loci, with 4 more loci validated (*CD2AP*, *PTK2B*, *SORL1*, and *SLC24A4*).^13^ The well‐replicated results showed the good performance of VEGAS2 for gene‐based analysis. For the full results, see Supporting Information Table S1.

For IS, no genes of gene‐wide significance were recognized (*P* < 2.28E−06 for 21,913 gene tests), while two genes surpassed a loose significance threshold (*P* < 1.00E−04): *ZYX *(*P* = 2.70E−05) and *NCR3LG1* *P* = 7.30E−05).

### 3.2 Gene‐based testing for shared genes between AD and IS

After overlapping 1915 AD genes and 1288 IS genes with nominal significance, 130 shared genes remained for further Fisher's meta‐analysis. We identified 17 genes reaching the significance threshold at *P*_combined_ < 1.92E−4 [0.05/(2*130)] (Table 1), some of which were located in adjacent positions, that is, *ZYX/EPHA1* at 7q34‐7q35, *EFTUD1/FAM154B* at 15q25.2, *YDJC/UBE2L3* and *SLC2A11* at 22q11, *HECTD4/OAS2 *at 12q24.13, and *ANKHD1‐EIF4EBP3/ANKHD1* at 5q31.3. The latter was regarded as one gene as they were likely to arise from the same SNPs signals as *ANKHD1‐EIF4EBP3* is a fusion transcript of *ANKHD1 *and its downstream *EIF4EBP3*. It's worth noting that *ZYX* and *EFTUD1* even reached *P* < 0.001 in both AD and IS datasets. For the full results, refer to Supporting Information Table S2.

**Table 1:** Gene‐based association results from gene‐based meta‐analyses of Alzheimer's disease (AD) and ischemic stroke (IS)

Region	Gene	AD			IS			*P*_combined_
		*P *value	Top‐SNP	Top‐SNP *P *value	*P* value	Top‐SNP	Top‐SNP *P* value	
7q34	***ZYX***	**5.10E−04**	rs6464548	1.62E**−**06	**2.70E−05**	rs11772895	3.39E**−**06	2.63E**−**07
7q34**‐**q35	*EPHA1*	1.00E**−**06	rs10808026	1.42E**−**11	3.64E**−**02	rs11762334	7.16E**−**05	6.60E**−**07
15q25.2	***EFTUD1***	**1.09E−04**	rs905450	2.82E**−**06	**8.24E−04**	rs151045855	2.17E**−**04	1.55E**−**06
11q12.2	*MS4A4A*	1.50E**−**05	rs55777218	3.94E**−**12	4.21E**−**02	rs115739426	5.29E**−**03	9.65E**−**06
22q11.21	*YDJC*	2.01E**−**04	rs2298428	7.28E**−**05	1.00E**−**02	rs3747093	2.87E**−**03	2.84E**−**05
22q11.21	*UBE2L3*	2.72E**−**04	rs12168746	8.82E**−**05	7.74E**−**03	rs738129	3.95E**−**03	2.96E**−**05
8q22.3	*PABPC1*	4.76E**−**03	rs1693547	9.19E**−**04	9.00E**−**04	rs3104313	1.94E**−**04	5.72E**−**05
16p12.2	*RRN3P1*	1.56E**−**03	rs12600118	3.92E**−**05	3.62E**−**03	rs4017431	8.17E**−**04	7.39E**−**05
15q25.2	*FAM154B*	2.56E**−**04	rs28522807	2.86E**−**04	2.56E**−**02	rs117388641	1.55E**−**03	8.48E**−**05
17q25.1	*SLC16A5*	7.22E**−**04	rs8078881	2.32E**−**04	1.23E**−**02	rs12943414	1.70E**−**03	1.12E**−**04
12q24.13	*HECTD4*	3.68E**−**02	rs147910225	2.31E**−**04	2.70E**−**04	rs10850034	2.49E**−**05	1.24E**−**04
5q31.3	*ANKHD1* **‐** *EIF4EBP3*	8.61E**−**04	rs78028717	2.52E**−**04	1.27E**−**02	rs801459	1.39E**−**03	1.36E**−**04
8p23.1	*PINX1*	1.23E**−**03	rs7014168	3.60E**−**04	9.20E**−**03	rs12676417	6.40E**−**04	1.40E**−**04
22q11.23	*SLC2A11*	1.12E**−**03	rs5760076	3.91E**−**05	1.06E**−**02	rs73158776	2.17E**−**03	1.47E**−**04
12q24.13	*OAS2*	2.50E**−**04	rs1635142	4.27E**−**05	4.96E**−**02	rs929291	8.24E**−**03	1.52E**−**04
5q31.3	*ANKHD1*	1.18E**−**03	rs78028717	2.52E**−**04	1.06E**−**02	rs2108446	1.85E**−**03	1.54E**−**04
6p21.1	*TREM2*	1.52E**−**03	rs6933067	1.07E**−**03	9.50E**−**03	rs7748513	4.34E**−**03	1.75E**−**04

Top‐SNP, most significant SNP from each gene. Significantly associated genes whose *P*‐values in both phenotypes were below 0.001 were shown in bold.

### 3.3 Gene expression analyses of shared genes

To validate the relevance of the 16 AD‐IS genes*, *we evaluated their expressions in brains or peripheral blood of AD or IS cases versus controls. Importantly, *MS4A4A* and *TREM2*, two established AD‐susceptibility genes, showed remarkable alterations in the spleens and brains afflicted by IS, respectively. A comparison of differentially expressed genes in each dataset was presented in Table 2.

**Table 2:** Expression changes of shared genes in distinct expression datasets for Alzheimer's disease (AD) and ischemic stroke (IS)

Gene	AD		IS		
	GSE44770	GSE48350	GSE55260	GSE16561	GSE70841
	PFC	Hippocampus	Brain	Peripheral blood	Spleen
*ZYX*	2.41E**−**01	2.13E**−**01	**8.43E−04**	**3.38E−04**	8.08E**−**02
*EPHA1*	6.12E**−**01	4.26E**−**02	9.48E**−**01	**1.22E−03**	9.23E**−**01
*EFTUD1*	2.48E**−**01	4.15E**−**02	4.79E**−**02	5.31E**−**02	8.60E**−**02
*MS4A4A*	**1.08E−24**	3.87E**−**03**	—	**2.73E−05**	**1.57E−05** *
*YDJC*	—	**1.14E−04**	7.58E**−**03	2.17E**−**01	4.61E**−**01
*UBE2L3*	**3.24E−20**	4.45E**−**01	7.32E**−**01	5.68E**−**02	**1.83E−03**
*PABPC1*	**1.90E−11**	**2.99E−04**	**4.17E−05** *	7.40E**−**02	5.27E**−**01
*RRN3P1*	—	2.69E**−**01	—	—	—
*FAM154B*	—	3.14E**−**01	5.54E**−**02	8.80E**−**01	2.55E**−**01
*SLC16A5*	**4.93E−10**	3.15E**−**01	1.16E**−**01	3.47E**−**02	—
*HECTD4*	**8.74E−16**	**5.17E−05**	—	—	—
*ANKHD1* **‐** *EIF4EBP3*	4.30E**−**01	2.05E**−**01	9.75E**−**02	4.15E**−**02	—
*PINX1*	**2.46E−04**	3.13E**−**01	2.03E**−**01	8.44E**−**01	—
*SLC2A11*	**1.41E−11**	5.39E**−**03	—	**2.55E−03**	—
*OAS2*	1.13E**−**01	1.46E**−**01	1.29E**−**01	2.04E**−**01	—
*TREM2*	**6.80E−20**	1.34E**−**01	**9.29E−06** *	5.37E**−**01	7.10E**−**01
Significance threshold	3.85E**−**03	3.13E**−**03	3.85E**−**03	3.57E**−**03	5.56E**−**03

GEO accession, GSE44772, GSE48350, GSE55260, GSE16561 and GSE70841; PFC, dorsolateral prefrontal cortex; “—”, (no data) meant that the transcripts of this gene did not exist in corresponding datasets. Bolded *P*‐values of genes achieved Bonferroni‐corrected significance, adjusted for the number of shared genes present in each expression dataset (0.05/n, n ≤ 16).

* Gene expression level showing more than 2 folds upregulation or downregulation compared to controls.

** Genes near the significance threshold.

AD‐associated expression profiles from the PFC region (Supporting Information Table S3A) revealed that 8 genes were differentially expressed, with *MS4A4A, UBE2L3,* *TREM2,* and *HECTD4* being the most significant, while just 3 genes’ expression levels (*HECTD4, YDJC, *and *PABPC1*) were altered in the hippocampal regions compared with control brains (Supporting Information Table S3B). In peri‐infarcted rat brains (Supporting Information Table S4A), we observed expression changes of 4 genes (*TREM2,* *PABPC1,* *ZYX*, and *YDJC*). Notably, *TREM2 *showed nearly 4‐fold upregulation versus controls (log2‐fold change = 1.99). Transcriptional changes of four genes (*MS4A4A*, *ZYX*, *EPHA1 *and SLC2A11) were present in the peripheral blood of IS patients (Supporting Information Table S4B). Particularly, differential expression of *MS4A4A* was even more evident in mouse spleens, with over 5‐fold increase (log2‐fold change = 2.37; Supporting Information Table S4C). It's necessary to point out that, however, the transcriptional data from rat brains and mouse spleens were not compelling enough due to the small sample size. More robust transcriptional datasets are needed to further validate the candidate genes.

## 4 DISCUSSION

Different from conventional SNP‐based GWAS studies, our research used VEGAS2 gene‐based association test to detect pleiotropic genes jointly associated with AD and IS. To avoid the problem of joint effects arising from a dominant association with one single disease, we focused on shared genes with nominal significance in both AD and IS (*P* < 0.05). By this criterion, 16 genes survived the stringent Bonferroni correction. Next, they were screened for differential expression in AD and IS cases versus controls. To search for supportive evidence for their relevance to AD and IS, we paid extra attention to the molecular functions of these genes.

### 4.1 *ZYX *and *EPHA1* gene at 7q34‐7q35

*Zyxin* (*ZYX*) encodes a zinc‐binding adaptor protein that translocates from focal adhesions to the nucleus to conduct signal transduction and modulate gene expression. Recently, zyxin has been identified as a novel target of Aβ metabolism in AD.^26^ Besides, Zyxin is a novel interacting partner of SIRT1^27^ that is protective against aging‐associated pathologies like AD^28^ and IS.^29^ Here, *ZYX* was jointly associated with AD and IS (*P*_combined_ = 2.63E−07) with *P* < 0.001 in each disease. Of note was that *ZYX *showed the most significant association (*P* = 2.70E−05) in the gene‐based analysis of IS.

*EPHA1 *is an established risk locus of AD, and our gene‐based analysis confirmed its gene‐wide association with AD (*P* = 1.00E−06). Ephrin type‐A receptor 1 (EPHA1) belongs to the eph receptor subfamily that is the largest family of receptor tyrosine kinases, mediating axonal guidance, synaptic plasticity, and cell‐to‐cell communication in the central nervous system (CNS).^30^, ^31^ Moreover, EPHA1 can modulate leukocyte extravasation, chemotaxis, and inflammatory cell migration.^32^, ^33^, ^34^ Indeed, ample evidence has implicated the involvement of *EPHA*1, as well as *MS4A4A* and *TREM2* listed below, in the immune module of AD.^22^, ^35^, ^36^ As previously described,^37^ we observed no aberrant *EPHA1* expression in the PFC region of AD patients, neither in the hippocampus.

### 4.2 *MS4A4A *gene at 11q12.2

*MS4A4A* is a strong AD candidate gene 38 within the membrane‐spanning 4‐domains subfamily A (*MS4A*) gene cluster, an established risk locus for AD.^13^, ^39^, ^40^ It is predominantly expressed in immune cells, including resident microglia in the CNS. Following microglial activation, *MS4A4A* shows increased expression.^41^ Similarly, *MS4A4A* is upregulated in activated dendritic cells (DCs) and M1 macrophages, while not detected in immature DCs and M2 macrophages.^42^ Here, *MS4A4A* showed significantly altered expression in the PFC region of AD brains (*P* = 1.08E−24), yet displayed an expression alteration of suggestive evidence in the hippocampal region (*P* = 3.87E−03).

Notably, *MS4A4A* was also differentially expressed in peripheral blood of IS patients (*P* = 2.73E−05) and in mouse spleens after IS with over 5‐fold upregulation (*P* = 1.57E−05; log2‐fold change = 2.37). Following IS, increased incidence of infections occurs, mainly in the form of pneumonia and urinary tract infections.^43^, ^44^ The underlying mechanism is insufficient antigen‐presentation of monocytes/macrophages and DCs in peripheral immune organs, resulting from downregulation of MHC class II and co‐stimulatory molecules and remarkable reduction of proinflammatory cytokines.^45^ Herein, we speculated that the pronounced upregulation of *MS4A4A *in the spleen following IS might reflect a phenotypic switch of monocytes from the proinflammatory M1 phenotype to the anti‐inflammatory M2 phenotype.

### 4.3 *UBE2L3*, *YDJC *and *SLC2A11* genes at 22q11

Ubiquitin‐conjugating enzyme E2 L3 *(UBE2L3)* and *YDJC* are located at 22q11.21. The genetic relationship between the 22q11.21 region and multiple autoimmune diseases has been extensively elucidated.^46^, ^47^, ^48^ Besides, SNPs near *YDJC* are suggested to be a pleiotropic locus between AD and Crohn disease.^49^ Lately, UBE2L3 has been identified as a hub gene in the gene regulatory networks of AD.^50^

*UBE2L3* encodes an E2 ubiquitin‐conjugating enzyme. Through its action on ubiquitination in NF‐κB signaling, UBE2L3 promotes NF‐κB activation, thus mediates its link with numerous autoimmune diseases.^51^, ^52^, ^53^ Moreover, UBE2L3 modulates pro‐IL‐1β processing and mature IL‐1β secretion,^54^ the deregulation of which pronouncedly intensifies neuronal damage in both AD and IS.^55^, ^56^ In addition, UBE2L3 directly interacts with the parkin protein, a ubiquitin‐protein ligase that is protective against not only neurodegenerative diseases,^57^, ^58^, ^59^ but also cerebral ischemia‐reperfusion injury.^60^ Nonetheless, there is no conclusive evidence to date demonstrating a causative link between *UBE2L3* and AD or IS. The function of *YDJC* remains largely obscure. Solute‐carrier 2A family member 11 (*SLC2A11*) encodes GLUT11, a fructose‐transporting protein that might participate in fructose consumption in the CNS.^61^

### 4.4 *PABPC1 *gene at 8q22.3

Poly(A) binding protein cytoplasmic 1 (PABPC1), one type of RNA‐binding proteins, is a central component of cytoplasmic stress granules comprising proteins and mRNAs stalled at the translation initiation step.^62^ Pathological stress granules play crucial roles in neurodegenerative disorders,^63^, ^64^ also in brain ischemia.^65^ Moreover, abnormal cytoplasmic inclusions of PABPC1 have been observed in human ALS spinal cord neurons.^66^, ^67^ In the present work, *PABPC1* was differentially expressed in both the PFC (*P* = 1.90E−11) and hippocampal regions (*P* = 2.99E−04) of AD. Evidently in IS brains, its expression was significantly enhanced (log2‐fold change = 1.19) compared to control subjects (Supporting Information Table S4A).

### 4.5 *HECTD4* and *OAS2* gene at 12q24

Mounting GWAS studies have demonstrated the pleiotropic effects of 12q24 locus on type 1 diabetes,^68^, ^69^ celiac disease,^70^ coronary artery disease 71, 72, 73 and a number of cardiovascular risk factors, including hypertension,^72^, ^74^, ^75^ cholesterol levels,^72^, ^76^ whist‐hip ration,^77^ and glycemia.^78^ Importantly, the 12q24 region has been suggested to be a risk locus of IS.^79^ Recently, SNPs near *HECTD4* are shown to be associated with memory performance.^80^ HECT domain E3 ubiquitin‐protein ligase 4 (HECTD4) is a E3 ubiquitin‐protein ligase. HECT‐type E3s can function with UBE2L3 discussed above in the ubiquitin system.^81^ Here, *HECTD4* was differentially expressed in both AD's PFC (*P* = 8.74E−16) and hippocampal (*P* = 1.73E−03) regions compared to controls. Regrettably, the transcripts of *HECTD4* were not detected in any expression datasets of IS. 2'‐5'‐oligoadenylate synthetase 2 (*OAS2*) gene, together with neighboring *OAS1* and *OAS3* gene, encodes enzymes participating in innate immunity response to viral infection.^82^

### 4.6 *PINX1* gene at 8p23.1

PIN2 (TERF1) interacting telomerase inhibitor 1 (PINX1) protein is a potent telomerase inhibitor,^83^ and a microtubule‐binding protein essential for chromosome segregation in mitosis.^84^ In addition to its biological significance in various cancers,^85^ *PINX1* gene is associated with subclinical cardiovascular events like carotid intima media thickness,^86^ blood lipids,^87^, ^88^ and involved in AD as a potential interactor of Aβ.^89^

### 4.7 *TREM2 *gene at 6p21.1

Triggering receptor expressed on myeloid cells‐2 (*TREM2*) is highly expressed on microglia as an innate immune receptor involved in phagocytosis, clearance of damaged neurons, and inhibition of the microglial proinflammatory response.^90^ Mutation of rare variants in *TREM2 *confers a substantial increase in AD risk,^91^, ^92^, ^93^ which has been experimentally proved.^94^, ^95^ *TREM2* has been indicated to be upregulated and participate in ischemic brain damage by modulating microglial phenotypes despite conflicting findings.^96^, ^97^, ^98^, ^99^, ^100^ Here, *TREM2* was shared by AD and IS with individual *P* = 0.0015 and *P* = 0.0095, respectively. Since rare variants (MAF < 0.01) were excluded from the GWAS panels of both AD and IS*, *we assumed that the true joint association signal of *TREM2 *with AD and IS might be stronger than we observed. *TREM2* transcriptional changes were observed in the PFC region (*P* = 6.80E−20) of AD, and in rat peri‐infarcted brains (*P* = 9.29E−06). Expectedly, the latter showed nearly 4‐fold increased expression for *TREM2* (log2‐fold change = 1.99; Supporting Information Table S4A).

### 4.8 Others

Though we found that the following genes (ie *EFTUD1*/*FAM154B* at 15q25.2, *RRN3P1*, *SLC16A5*, and *ANKHD1‐EIF4EBP3*) were common association signals for AD and IS in terms of bioinformatics, less is known about their biological roles due to lack of overwhelming evidence related to AD or IS.

Based on the concise discussion about their biological significance, partial pleotropic genes underlying AD and IS were prioritized, including*ZYX*, *EPHA1*, *MS4A4A*, *UBE2L3*, *PABPC1*, *HECTD4*, *PINX1*, and *TREM2*. Consistent with our previous findings from the pathway‐oriented perspective,^101^ we once again highlighted the critical roles of neuroinflammation in the development and progression of AD and IS, since half of them (*EPHA1*, *MS4A4A*, *UBE2L3* and *TREM2*) were engaged in immune signaling.

Although gene‐based tests increase the power to detect disease‐associated genes harboring multiple associated variants, they do have limitations. Firstly, the VEGAS test is prone to underestimating effects of low‐frequency SNPs correlated with few SNPs in LD blocks,^102^ but may unable to distinguish the truly casual genes from several adjacent ones colocalizing in one significant locus. Secondly, genes revealed by positional proximity to significant variants are not necessarily the casual ones for disease pathogenesis. In complex diseases, significant variants are mostly located in intronic/intergenic areas, presumably regulating gene expression, including acting on distant genes. Next, we leveraged the GEO dataset to estimate the expression alteration of candidate genes in disease‐related tissues, the reliability of which largely relied on the raw data, for instance, the size of tissue samples in the original studies. Further powerful approaches and sophisticated functional interpretation analyses are warranted to prioritize causal genes. Moreover, being pathologically and genetically heterogeneous,^103^ IS has different etiological subtypes (ie large vessel disease, cardioembolic stroke, and small vessel disease, undetermined and other). Here, we just surveyed the genetic link between AD and overall IS.

## 5 CONCLUSIONS

We presented a gene‐based strategy that corroborates shared candidate genes between AD and IS, with gene expression analysis ensued, which provided a typical example of how genetic studies could add to biological understanding of cross‐trait etiology. Literature mining supported the potential association of partial novel candidate genes with both AD and IS. Our findings yielded mechanistic insights into the common pathogenesis underlying AD and IS, predominantly involving the immune system, and might suggest common intervention targets. More importantly, our findings should encourage more studies to verify the involvement of these candidates in AD and IS and interpret the exact molecular mechanisms of action.

## CONFLICT OF INTEREST

The authors declare no conflict of interest.

## References

[1] Kassebaum NJ , Arora M , Barber RM , et al. Global, regional, and national disability‐adjusted life‐years (DALYs) for 315 diseases and injuries and healthy life expectancy (HALE), 1990‐2015: a systematic analysis for the Global Burden of Disease Study 2015. The Lancet. 2016;388(10053):1603‐1658. 10.1016/S0140-6736(16)31460-X PMC5388857 27733283

[2] Wang H , Naghavi M , Allen C , et al. Global, regional, and national life expectancy, all‐cause mortality, and cause‐specific mortality for 249 causes of death, 1980‐2015: a systematic analysis for the Global Burden of Disease Study 2015. The Lancet. 2016;388(10053):1459‐1544. 10.1016/S0140-6736(16)31012-1 PMC5388903 27733281

[3] Langhorne P , Bernhardt J , Kwakkel G . Stroke rehabilitation. Lancet. 2011;377(9778):1693‐1702. 21571152 10.1016/S0140-6736(11)60325-5

[4] Tolppanen AM , Lavikainen P , Solomon A , Kivipelto M , Soininen H , Hartikainen S . Incidence of stroke in people with Alzheimer disease: a national register‐based approach. Neurology. 2013;80(4):353‐358. 23284063 10.1212/WNL.0b013e31827f08c5

[5] Chi NF , Chien LN , Ku HL , Hu CJ , Chiou HY . Alzheimer disease and risk of stroke: a population‐based cohort study. Neurology. 2013;80(8):705‐711. 23303851 10.1212/WNL.0b013e31828250af

[6] Gamaldo A , Moghekar A , Kilada S , Resnick SM , Zonderman AB , O'Brien R . Effect of a clinical stroke on the risk of dementia in a prospective cohort. Neurology. 2006;67(8):1363‐1369. 17060561 10.1212/01.wnl.0000240285.89067.3f

[7] Dong S , Maniar S , Manole MD , Sun D . Cerebral hypoperfusion and other shared brain pathologies in ischemic stroke and Alzheimer's disease. Transl Stroke Res. 2018;9(3):238‐250. 28971348 10.1007/s12975-017-0570-2 PMC9732865

[8] Gorelick PB , Scuteri A , Black SE , et al. Vascular contributions to cognitive impairment and dementia: a statement for healthcare professionals from the american heart association/american stroke association. Stroke. 2011;42(9):2672‐2713. 21778438 10.1161/STR.0b013e3182299496 PMC3778669

[9] Pluta R , Jabłoński M , Ułamek‐Kozioł M , et al. Sporadic Alzheimer's disease begins as episodes of brain ischemia and ischemically dysregulated Alzheimer's disease genes. Mol Neurobiol. 2013;48(3):500‐515. 23519520 10.1007/s12035-013-8439-1 PMC3825141

[10] Salminen A , Kauppinen A , Kaarniranta K . Hypoxia/ischemia activate processing of Amyloid Precursor Protein: impact of vascular dysfunction in the pathogenesis of Alzheimer's disease. J Neurochem. 2017;140(4):536‐549. 27987381 10.1111/jnc.13932

[11] Bi M , Gladbach A , van Eersel J , et al. Tau exacerbates excitotoxic brain damage in an animal model of stroke. Nat Commun. 2017;8(1):473. 28883427 10.1038/s41467-017-00618-0 PMC5589746

[12] Tuo Q‐z , Lei P , Jackman Ka , et al. Tau‐mediated iron export prevents ferroptotic damage after ischemic stroke. Mol Psychiatry. 2017;22(11):1520‐1530. 28886009 10.1038/mp.2017.171

[13] Lambert J‐C , Ibrahim‐Verbaas CA , Harold D , et al. Meta‐analysis of 74,046 individuals identifies 11 new susceptibility loci for Alzheimer's disease. Nat Genet. 2013;45(12):1452‐1458. 24162737 10.1038/ng.2802 PMC3896259

[14] Jansen IE , Savage JE , Watanabe K , et al. Genome‐wide meta‐analysis identifies new loci and functional pathways influencing Alzheimer's disease risk. Nat Genet. 2019;51(3):404‐413. 30617256 10.1038/s41588-018-0311-9 PMC6836675

[15] Malik R , Traylor M , Pulit SL , et al. Low‐frequency and common genetic variation in ischemic stroke: The METASTROKE collaboration. Neurology. 2016;86(13):1217‐1226. 26935894 10.1212/WNL.0000000000002528 PMC4818561

[16] Malik R , Chauhan G , Traylor M , et al. Multiancestry genome‐wide association study of 520,000 subjects identifies 32 loci associated with stroke and stroke subtypes. Nat Genet. 2018;50(4):524‐537. 29531354 10.1038/s41588-018-0058-3 PMC5968830

[17] Traylor M , Adib‐Samii P , Harold D , et al. Shared genetic contribution to Ischaemic Stroke and Alzheimer's Disease. Ann Neurol. 2016;79(5):739‐747. 26913989 10.1002/ana.24621 PMC4864940

[18] Boyle EA , Li YI , Pritchard JK . An expanded view of complex traits: from polygenic to omnigenic. Cell. 2017;169(7): 1177‐1186. 28622505 10.1016/j.cell.2017.05.038 PMC5536862

[19] Mishra A , Macgregor S . VEGAS2: Software for more flexible gene‐based testing. Twin Res Hum Genet. 2015;18(1):86‐91. 25518859 10.1017/thg.2014.79

[20] Liu JZ , Mcrae AF , Nyholt DR , et al. A versatile gene‐based test for genome‐wide association studies. Am J Hum Genet. 2010;87(1):139‐145. 20598278 10.1016/j.ajhg.2010.06.009 PMC2896770

[21] Begum F , Ghosh D , Tseng GC , Feingold E . Comprehensive literature review and statistical considerations for GWAS meta‐analysis. Nucleic Acids Res. 2012;40(9):3777‐3784. 22241776 10.1093/nar/gkr1255 PMC3351172

[22] Zhang B , Gaiteri C , Bodea L‐G , et al. Integrated systems approach identifies genetic nodes and networks in late‐onszheimer's disease. Cell. 2013;153(3):707‐720. 23622250 10.1016/j.cell.2013.03.030 PMC3677161

[23] Berchtold NC , Coleman PD , Cribbs DH , Rogers J , Gillen DL , Cotman CW . Synaptic genes are extensively downregulated across multiple brain regions in normal human aging and Alzheimer's disease. Neurobiol Aging. 2013;34(6):1653‐1661. 23273601 10.1016/j.neurobiolaging.2012.11.024 PMC4022280

[24] Buga AM , Margaritescu C , Scholz CJ , Radu E , Zelenak C , Popa‐Wagner A . Transcriptomics of post‐stroke angiogenesis in the aged brain. Front Aging Neurosci. 2014;6:44. 24672479 10.3389/fnagi.2014.00044 PMC3957426

[25] Barr Tl , Conley Y , Ding J , et al. Genomic biomarkers and cellular pathways of ischemic stroke by RNA gene expression profiling. Neurology. 2010;75(11):1009‐1014. 20837969 10.1212/WNL.0b013e3181f2b37f PMC2942033

[26] Lanni C , Necchi D , Pinto A , et al. Zyxin is a novel target for beta‐amyloid peptide: characterization of its role in Alzheimer's pathogenesis. J Neurochem. 2013;125(5):790‐799. 23330981 10.1111/jnc.12154

[27] Fujita Y , Yamaguchi A , Hata K , Endo M , Yamaguchi N , Yamashita T . Zyxin is a novel interacting partner for SIRT1. BMC Cell Biol. 2009;10:6. 19173742 10.1186/1471-2121-10-6 PMC2642761

[28] Herskovits AZ , Guarente L . SIRT1 in neurodevelopment and brain senescence. Neuron. 2014;81(3):471‐483. 24507186 10.1016/j.neuron.2014.01.028 PMC4040287

[29] Yang Y , Duan W , Li Y , et al. New role of silent information regulator 1 in cerebral ischemia. Neurobiol Aging. 2013;34(12):2879‐2888. 23855981 10.1016/j.neurobiolaging.2013.06.008

[30] Martinez A , Otal R , Sieber BA , Ibanez C , Soriano E . Disruption of ephrin‐A/EphA binding alters synaptogenesis and neural connectivity in the hippocampus. Neuroscience. 2005;135(2):451‐461. 16112477 10.1016/j.neuroscience.2005.06.052

[31] Lai KO , Ip NY . Synapse development and plasticity: roles of ephrin/Eph receptor signaling. Curr Opin Neurobiol. 2009;19(3):275‐283. 19497733 10.1016/j.conb.2009.04.009

[32] Ivanov AI , Romanovsky AA . Putative dual role of ephrin‐Eph receptor interactions in inflammation. IUBMB Life. 2006;58(7):389‐394. 16801213 10.1080/15216540600756004

[33] Aasheim HC , Delabie J , Finne EF . Ephrin‐A1 binding to CD4+ T lymphocytes stimulates migration and induces tyrosine phosphorylation of PYK2. Blood. 2005;105(7):2869‐2876. 15585656 10.1182/blood-2004-08-2981

[34] Ahmad S , Bannister C , van der Lee SJ , et al. Disentangling the biological pathways involved in early features of Alzheimer's disease in the Rotterdam Study. Alzheimers Dement. 2018;14(7):848‐857. 29494809 10.1016/j.jalz.2018.01.005

[35] Heneka MT , Golenbock DT , Latz E . Innate immunity in Alzheimer's disease. Nat Immunol. 2015;16(3):229‐236. 25689443 10.1038/ni.3102

[36] Karch CM , Cruchaga C , Goate A . Alzheimer’s Disease Genetics: From the bench to the clinic. Neuron. 2014;83(1):11‐26. 24991952 10.1016/j.neuron.2014.05.041 PMC4120741

[37] Karch CM , Jeng AT , Nowotny P , Cady J , Cruchaga C , Goate AM . Expression of Novel Alzheimer’s disease risk genes in control and Alzheimer’s disease brains. PLoS ONE. 2012;7(11):e50976. 23226438 10.1371/journal.pone.0050976 PMC3511432

[38] Allen M , Zou F , Chai Hs , et al. Novel late‐onszheimer disease loci variants associate with brain gene expression. Neurology. 2012;79(3):221‐228. 22722634 10.1212/WNL.0b013e3182605801 PMC3398432

[39] Hollingworth P , Harold D , Sims R , et al. Common variants at ABCA7, MS4A6A/MS4A4E, EPHA1, CD33 and CD2AP are associated with Alzheimer's disease. Nat Genet. 2011;43(5):429‐435. 21460840 10.1038/ng.803 PMC3084173

[40] Naj AC , Jun G , Beecham GW , et al. Common variants at MS4A4/MS4A6E, CD2AP, CD33 and EPHA1 are associated with late‐onszheimer's disease. Nat Genet. 2011;43(5):436‐441. 21460841 10.1038/ng.801 PMC3090745

[41] Kofler J , Bissel S , Wiley C , Stauffer M , Murdoch G . Differential microglial expression of new Alzheimer's disease associated genes MS4A4A and MS4A6A. Alzheimers Dement. 2012;8(4):P253‐P253.

[42] Sanyal R , Polyak MJ , Zuccolo J , et al. MS4A4A: a novel cell surface marker for M2 macrophages and plasma cells. Immunol Cell Biol. 2017;95(7):611‐619. 28303902 10.1038/icb.2017.18

[43] Rohweder G , Ellekjaer H , Salvesen O , Naalsund E , Indredavik B . Functional outcome after common poststroke complications occurring in the first 90 days. Stroke. 2015;46(1):65‐70. 25395415 10.1161/STROKEAHA.114.006667

[44] Meisel C , Schwab JM , Prass K , Meisel A , Dirnagl U . Central nervous system injury‐induced immune deficiency syndrome. Nat Rev Neurosci. 2005;6(10):775‐786. 16163382 10.1038/nrn1765

[45] Chamorro A , Meisel A , Planas AM , Urra X , van de Beek D , Veltkamp R . The immunology of acute stroke. Nat Rev Neurol. 2012;8(7):401‐410. 22664787 10.1038/nrneurol.2012.98

[46] Han J‐W , Zheng H‐F , Cui Y , et al. Genome‐wide association study in a Chinese Han population identifies nine new susceptibility loci for systemic lupus erythematosus. Nat Genet. 2009;41(11):1234‐1237. 19838193 10.1038/ng.472

[47] Zhernakova A , Stahl EA , Trynka G , et al. Meta‐analysis of genome‐wide association studies in celiac disease and rheumatoid arthritis identifies fourteen non‐HLA shared loci. PLoS Genet. 2011;7(2):e1002004. 21383967 10.1371/journal.pgen.1002004 PMC3044685

[48] Ellinghaus D , Ellinghaus E , Nair R , et al. Combined analysis of genome‐wide association studies for crohn disease and psoriasis identifies seven shared susceptibility loci. Am J Hum Genet. 2012;90(4):636‐647. 22482804 10.1016/j.ajhg.2012.02.020 PMC3322238

[49] Yokoyama JS , Wang Y , Schork AJ , et al. Association between genetic traits for immune‐mediated diseases and alzheimer disease. JAMA Neurol. 2016;73(6):691‐697. 27088644 10.1001/jamaneurol.2016.0150 PMC4905783

[50] Kawalia SB , Raschka T , Naz M , de Matos SR , Senger P , Hofmann‐Apitius M . Analytical strategy to prioritize Alzheimer’s disease candidate genes in gene regulatory networks using public expression data. J Alzheimers Dis. 2017;59(4):1237‐1254. 28800327 10.3233/JAD-170011 PMC5611835

[51] Fu B , Li S , Wang L , Berman MA , Dorf ME . The ubiquitin conjugating enzyme UBE2L3 regulates TNFα‐induced linear ubiquitination. Cell Res. 2014;24(3):376‐379. 24060851 10.1038/cr.2013.133 PMC3945881

[52] Lewis MJ , Vyse S , Shields AM , et al. UBE2L3 polymorphism amplifies NF‐kappaB activation and promotes plasma cell development, linking linear ubiquitination to multiple autoimmune diseases. Am J Hum Genet. 2015;96(2):221‐234. 25640675 10.1016/j.ajhg.2014.12.024 PMC4320258

[53] Lewis M , Vyse S , Shields A , et al. Effect of UBE2L3 genotype on regulation of the linear ubiquitin chain assembly complex in systemic lupus erythematosus. Lancet. 2015;385(Suppl 1):S9. 10.1016/S0140-6736(15)60324-5 26312912

[54] Eldridge M , Sanchez‐Garrido J , Hoben GF , Goddard PJ , Shenoy AR . The Atypical Ubiquitin E2 Conjugase UBE2L3 Is an Indirect Caspase‐1 Target and Controls IL‐1β Secretion by Inflammasomes. Cell Rep. 2017;18(5):1285‐1297. 28147281 10.1016/j.celrep.2017.01.015 PMC5300903

[55] Gelderblom M , Sobey CG , Kleinschnitz C , Magnus T . Danger signals in stroke. Ageing Res Rev. 2015;24(Pt A):77‐82. 26210897 10.1016/j.arr.2015.07.004

[56] Heneka MT , Kummer MP , Stutz A , et al. NLRP3 is activated in Alzheimer's disease and contributes to pathology in APP/PS1 mice. Nature. 2013;493(7434):674‐678. 23254930 10.1038/nature11729 PMC3812809

[57] Shimura H , Hattori N , Kubo S‐I , et al. Familial Parkinson disease gene product, parkin, is a ubiquitin‐protein ligase. Nat Genet. 2000;25(3):302‐305. 10888878 10.1038/77060

[58] Burns MP , Zhang L , Rebeck GW , Querfurth HW , Moussa CE . Parkin promotes intracellular Abeta1‐42 clearance. Hum Mol Genet. 2009;18(17):3206‐3216. 19483198 10.1093/hmg/ddp258 PMC2733820

[59] Zheng Q , Huang T , Zhang L , et al. Dysregulation of ubiquitin‐proteasome system in neurodegenerative diseases. Front Aging Neurosci. 2016;8:303. 28018215 10.3389/fnagi.2016.00303 PMC5156861

[60] Tang J , Hu Z , Tan J , Yang S , Zeng L . Parkin protects against oxygen‐glucose deprivation/reperfusion insult by promoting Drp1 degradation. Oxid Med Cell Longev. 2016;2016:8474303. 27597885 10.1155/2016/8474303 PMC5002297

[61] Izumi Y , Zorumski CF . Glial‐neuronal interactions underlying fructose utilization in rat hippocampal slices. Neuroscience. 2009;161(3):847‐854. 19362122 10.1016/j.neuroscience.2009.04.008 PMC2689788

[62] Bag J , Bhattacharjee RB . Multiple levels of post‐transcriptional control of expression of the poly (A)‐binding protein. RNA Biol. 2010;7(1):5‐12. 20009508 10.4161/rna.7.1.10256

[63] Wolozin B . Physiological protein aggregation run amuck: stress granules and the genesis of neurodegenerative disease. Discov Med. 2014;17(91):47‐52. 24411700 PMC4694572

[64] Dewey CM , Cenik B , Sephton CF , Johnson BA , Herz J , Yu G . TDP‐43 aggregation in neurodegeneration: are stress granules the key? Brain Res. 2012;1462:16‐25. 22405725 10.1016/j.brainres.2012.02.032 PMC3372581

[65] Ayuso MI , Martinez‐Alonso E , Regidor I , Alcazar A . Stress granule induction after brain ischemia is independent of eukaryotic translation initiation factor (eIF) 2alpha phosphorylation and is correlated with a decrease in eIF4B and eIF4E proteins. J Biol Chem. 2016;291(53):27252‐27264. 27836976 10.1074/jbc.M116.738989 PMC5207152

[66] Kim H‐J , Raphael AR , LaDow ES , et al. Therapeutic modulation of eIF2α‐phosphorylation rescues TDP‐43 toxicity in amyotrophic lateral sclerosis disease models. Nat Genet. 2014;46(2):152‐160. 24336168 10.1038/ng.2853 PMC3934366

[67] Fittschen M , Lastres‐Becker I , Halbach Mv , et al. Genetic ablation of ataxin‐2 increases several global translation factors in their transcript abundance but decreases translation rate. Neurogenetics. 2015;16(3):181‐192. 25721894 10.1007/s10048-015-0441-5 PMC4475250

[68] Burton PR , Clayton DG , Cardon LR , et al. Genome‐wide association study of 14,000 cases of seven common diseases and 3,000 shared controls. Nature. 2007;447(7145):661‐678. 17554300 10.1038/nature05911 PMC2719288

[69] Todd JA , Walker NM , Cooper JD , et al. Robust associations of four new chromosome regions from genome‐wide analyses of type 1 diabetes. Nat Genet. 2007;39(7):857‐864. 17554260 10.1038/ng2068 PMC2492393

[70] Hunt KA , Zhernakova A , Turner G , et al. Newly identified genetic risk variants for celiac disease related to the immune response. Nat Genet. 2008;40(4):395‐402. 18311140 10.1038/ng.102 PMC2673512

[71] Lu X , Wang L , Chen S , et al. Genome‐wide association study in Han Chinese identifies four new susceptibility loci for coronary artery disease. Nat Genet. 2012;44(8):890‐894. 22751097 10.1038/ng.2337 PMC3927410

[72] Kato N , Takeuchi F , Tabara Y , et al. Meta‐analysis of genome‐wide association studies identifies common variants associated with blood pressure variation in east Asians. Nat Genet. 2011;43(6):531‐538. 21572416 10.1038/ng.834 PMC3158568

[73] Soranzo N , Spector TD , Mangino M , et al. A genome‐wide meta‐analysis identifies 22 loci associated with eight hematological parameters in the HaemGen consortium. Nat Genet. 2009;41(11):1182‐1190. 19820697 10.1038/ng.467 PMC3108459

[74] Newton‐Cheh C , Johnson T , Gateva V , et al. Eight blood pressure loci identified by genome‐wide association study of 34,433 people of European ancestry. Nat Genet. 2009;41(6):666‐676. 19430483 10.1038/ng.361 PMC2891673

[75] Levy D , Ehret GB , Rice K , et al. Genome‐wide association study of blood pressure and hypertension. Nat Genet. 2009;41(6):677‐687. 19430479 10.1038/ng.384 PMC2998712

[76] Kim YJ , Go MJ , Hu C , et al. Large‐scale genome‐wide association studies in East Asians identify new genetic loci influencing metabolic traits. Nat Genet. 2011;43(10):990‐995. 21909109 10.1038/ng.939

[77] Cho YS , Go MJ , Kim YJ , et al. A large‐scale genome‐wide association study of Asian populations uncovers genetic factors influencing eight quantitative traits. Nat Genet. 2009;41(5):527‐534. 19396169 10.1038/ng.357

[78] Go MJ , Hwang J‐Y , Kim YJ , et al. New susceptibility loci in MYL2, C12orf51 and OAS1 associated with 1‐h plasma glucose as predisposing risk factors for type 2 diabetes in the Korean population. J Hum Genet. 2013;58(6):362‐365. 23575436 10.1038/jhg.2013.14

[79] Kilarski LL , Achterberg S , Devan WJ , et al. Meta‐analysis in more than 17,900 cases of ischemic stroke reveals a novel association at 12q24.12. Neurology. 2014;83(8):678‐685. 25031287 10.1212/WNL.0000000000000707 PMC4150131

[80] Ninomiya‐Baba M , Matsuo J , Sasayama D , et al. Association of body mass index‐related single nucleotide polymorphisms with psychiatric disease and memory performance in a Japanese population. Acta Neuropsychiatr. 2017;29(5):299‐308. 27923415 10.1017/neu.2016.66

[81] Wenzel DM , Lissounov A , Brzovic PS , Klevit RE . UbcH7 reactivity profile reveals Parkin and HHARI to be RING/HECT hybrids. Nature. 2011;474(7349):105‐108. 21532592 10.1038/nature09966 PMC3444301

[82] Field LL , Bonnevie‐Nielsen V , Pociot F , Lu S , Nielsen TB , Beck‐Nielsen H . OAS1 splice site polymorphism controlling antiviral enzyme activity influences susceptibility to type 1 diabetes. Diabetes. 2005;54(5):1588‐1591. 15855350 10.2337/diabetes.54.5.1588

[83] Zhou XZ , Lu KP . The Pin2/TRF1‐interacting protein PinX1 is a potent telomerase inhibitor. Cell. 2001;107(3):347‐359. 11701125 10.1016/s0092-8674(01)00538-4

[84] Yuan K , Li Na , Jiang K , et al. PinX1 is a novel microtubule‐binding protein essential for accurate chromosome segregation. J Biol Chem. 2009;284(34):23072‐23082. 19553660 10.1074/jbc.M109.001990 PMC2755713

[85] Zhang B , Bai Yx , Ma Hh , et al. Silencing PinX1 compromises telomere length maintenance as well as tumorigenicity in telomerase‐positive human cancer cells. Cancer Res. 2009;69(1):75‐83. 19117989 10.1158/0008-5472.CAN-08-1393

[86] Bis JC , Kavousi M , Franceschini N , et al. Meta‐analysis of genome‐wide association studies from the CHARGE consortium identifies common variants associated with carotid intima media thickness and plaque. Nat Genet. 2011;43(10):940‐947. 21909108 10.1038/ng.920 PMC3257519

[87] Willer CJ , Schmidt EM , Sengupta S , et al. Discovery and refinement of loci associated with lipid levels. Nat Genet. 2013;45(11):1274‐1283. 24097068 10.1038/ng.2797 PMC3838666

[88] Teslovich TM , Musunuru K , Smith AV , et al. Biological, clinical and population relevance of 95 loci for blood lipids. Nature. 2010;466(7307):707‐713. 20686565 10.1038/nature09270 PMC3039276

[89] Virok DP , Simon Dóra , Bozsó Z , et al. Protein array based interactome analysis of Amyloid‐β indicates an inhibition of protein translation. J Proteome Res. 2011;10(4):1538‐1547. 21244100 10.1021/pr1009096

[90] Colonna M . TREMs in the immune system and beyond. Nat Rev Immunol. 2003;3(6):445‐453. 12776204 10.1038/nri1106

[91] Guerreiro R , Wojtas A , Bras J , et al. TREM2 variants in Alzheimer's disease. N Engl J Med. 2013;368(2):117‐127. 23150934 10.1056/NEJMoa1211851 PMC3631573

[92] Jonsson T , Stefansson H , Steinberg S , et al. Variant of TREM2 associated with the risk of Alzheimer's disease. N Engl J Med. 2013;368(2):107‐116. 23150908 10.1056/NEJMoa1211103 PMC3677583

[93] Sims R , van der Lee SJ , Naj AC , et al. Rare coding variants in PLCG2, ABI3, and TREM2 implicate microglial‐mediated innate immunity in Alzheimer's disease. Nat Genet. 2017;49(9):1373‐1384. 28714976 10.1038/ng.3916 PMC5669039

[94] Wang Y , Cella M , Mallinson K , et al. TREM2 lipid sensing sustains the microglial response in an Alzheimer's disease model. Cell. 2015;160(6):1061‐1071. 25728668 10.1016/j.cell.2015.01.049 PMC4477963

[95] Ulland TK , Song WM , Huang SC , et al. TREM2 maintains microglial metabolic fitness in Alzheimer's disease. Cell. 2017;170(4):649‐663.e613. 28802038 10.1016/j.cell.2017.07.023 PMC5573224

[96] Sieber MW , Jaenisch N , Brehm M , et al. Attenuated inflammatory response in triggering receptor expressed on myeloid cells 2 (TREM2) knock‐out mice following stroke. PLoS ONE. 2013;8(1):e52982. 23301011 10.1371/journal.pone.0052982 PMC3536811

[97] Kawabori M , Kacimi R , Kauppinen T , et al. Triggering receptor expressed on myeloid cells 2 (TREM2) deficiency attenuates phagocytic activities of microglia and exacerbates ischemic damage in experimental stroke. J Neurosci. 2015;35(8):3384‐3396. 25716838 10.1523/JNEUROSCI.2620-14.2015 PMC4339351

[98] Zhai Q , Li F , Chen X , et al. Triggering receptor expressed on myeloid cells 2, a novel regulator of immunocyte phenotypes, Confers Neuroprotection by Relieving Neuroinflammation. Anesthesiology. 2017;127(1):98‐110. 28398927 10.1097/ALN.0000000000001628

[99] Kawabori M , Hokari M , Zheng Z , et al. Triggering receptor expressed on myeloid cells‐2 correlates to hypothermic neuroprotection in ischemic stroke. Ther Hypothermia Temp Manag. 2013;3(4):189‐198. 24380032 10.1089/ther.2013.0020 PMC3868297

[100] Wu R , Li X , Xu P , et al. TREM2 protects against cerebral ischemia/reperfusion injury. Mol Brain. 2017;10(1):20. 28592261 10.1186/s13041-017-0296-9 PMC5461720

[101] Cui P , Ma X , Li H , Lang W , Hao J . Shared biological pathways between Alzheimer’s disease and ischemic stroke. Front Neurosci. 2018;12(605). 10.3389/fnins.2018.00605 PMC6137293 30245614

[102] Huang H , Chanda P , Alonso A , Bader JS , Arking DE . Gene‐based tests of association. PLoS Genet. 2011;7(7):e1002177. 21829371 10.1371/journal.pgen.1002177 PMC3145613

[103] Markus HS , Bevan S . Mechanisms and treatment of ischaemic stroke–insights from genetic associations. Nat Rev Neurol. 2014;10(12):723‐730. 25348005 10.1038/nrneurol.2014.196

