# Supplementary material for: Shared genes between Alzheimer’s disease and ischemic stroke
Source: CNS Neurosci Ther. 2019 Mar 11;25(8):855–64. doi: 10.1111/cns.13117 (PMC6630005; doi:10.1111/cns.13117)
Supplement: Supplementary file 2 [file CNS-25-855-s002.docx]

Table S3 Gene expression changes in the PFC and hippocampal regions of AD brains

(A) AD-associated expression profile in the PFC region from GSE44772

| ID | *P*.Value | t | B | logFC | Gene |
| --- | --- | --- | --- | --- | --- |
| 10023804739 | **1.08E-24** | -11.55995 | 45.36121 | 3.74E-01 | ***MS4A4A*** |
| 10025903043 | **3.24E-20** | -10.13655 | 35.16143 | 9.42E-02 | ***UBE2L3*** |
| 10023826759 | **6.80E-20** | -10.03174 | 34.42893 | 2.58E-01 | ***TREM2*** |
| 10023810102 | **8.74E-16** | 8.650652 | 25.08898 | -1.11E-01 | ***HECTD4*** |
| 10025907596 | **1.41E-11** | 7.11337 | 15.56527 | -1.01E-01 | ***SLC2A11*** |
| 10023805421 | **1.90E-11** | -7.062794 | 15.27111 | 7.39E-02 | ***PABPC1*** |
| 10023822546 | **4.93E-10** | -6.498478 | 12.08347 | 8.58E-02 | ***SLC16A5*** |
| 10023830030 | **2.46E-04** | -3.723975 | -0.56705 | 5.43E-02 | ***PINX1*** |
| 10025906224 | 1.13E-01 | 1.593081 | -6.0372 | -1.31E-02 | *OAS2* |
| 10023815205 | 2.41E-01 | 1.175764 | -6.60996 | -1.15E-02 | *ZYX* |
| 10023848743 | 2.48E-01 | -1.157311 | -6.63136 | 5.78E-03 | *EFTUD1* |
| 10025908862 | 4.30E-01 | 0.790975 | -6.98664 | -6.21E-03 | *ANKHD1-EIF4EBP3* |
| 10025909326 | 6.12E-01 | 0.507934 | -7.17008 | -2.27E-03 | *EPHA1* |

Differentially expressed genes after Bonferroni correction (*P*<0.05/13) were in bold. PFC= dorsolateral prefrontal cortex, logFC=log2-fold change.

(B) AD-associated expression profile in the hippocampus from GSE48350

| ID | *P*.Value | t | B | logFC | Gene |
| --- | --- | --- | --- | --- | --- |
| 211034_s_at | **5.17E-05** | -4.340671 | 1.810081 | -0.369372 | ***HECTD4*** |
| 227042_at | **1.14E-04** | -4.113049 | 1.08889 | -0.362229 | ***YDJC*** |
| 215157_x_at | **2.99E-04** | 3.825679 | 0.210027 | 0.452411 | ***PABPC1*** |
| 224357_s_at | 3.87E-03 | 2.99742 | -2.088246 | 0.307252 | *MS4A4A* |
| 232167_at | 5.39E-03 | -2.881202 | -2.378709 | -0.325648 | *SLC2A11* |
| 218973_at | 4.15E-02 | -2.080214 | -4.127536 | -0.209002 | *EFTUD1* |
| 241482_at | 4.26E-02 | 2.068993 | -4.148651 | 0.227129 | *EPHA1* |
| 219725_at | 1.34E-01 | 1.517829 | -5.05944 | 0.473175 | *TREM2* |
| 228607_at | 1.46E-01 | 1.472334 | -5.123157 | 0.121788 | *OAS2* |
| 233292_s_at | 2.05E-01 | 1.280231 | -5.37213 | 0.231074 | *ANKHD1-EIF4EBP3* |
| 200808_s_at | 2.13E-01 | -1.256591 | -5.400499 | -0.155131 | *ZYX* |
| 221714_s_at | 2.69E-01 | 1.115093 | -5.559753 | 0.089264 | *RRN3P1* |
| 223907_s_at | 3.13E-01 | 1.017834 | -5.658627 | 0.076355 | *PINX1* |
| 1556158_at | 3.14E-01 | 1.015568 | -5.660827 | 0.086069 | *SAXO2* |
| 213590_at | 3.15E-01 | 1.012592 | -5.663709 | 0.11386 | *SLC16A5* |
| 200682_s_at | 4.45E-01 | -0.768029 | -5.872378 | -0.049874 | *UBE2L3* |
| Differentially expressed genes after Bonferroni correction (*P*<0.05/16) were in bold. *SAXO2* is the alias for *FAM154B*. logFC=log2-fold change. | | | | | |

Table S4 Gene expression changes in brain, peripheral blood and spleen of IS

(A) IS-associated expression profile in the rat brain from GSE55260

| ID | *P*.Value | t | B | logFC | Gene |
| --- | --- | --- | --- | --- | --- |
| 10926245 | **9.29E-06** | -13.37502 | 4.395764 | 1.988457 | ***Trem2*^†^** |
| 10903346 | **4.17E-05** | -10.36528 | 2.829364 | 1.191598 | ***Pabpc1*^†^** |
| 10855062 | **8.43E-04** | -6.072777 | -0.44982 | 0.88915 | ***Zyx*** |
| 10755790 | 7.58E-03 | 3.911921 | -2.873386 | -0.432322 | *Ydjc* |
| 10708379 | 4.79E-02 | -2.467253 | -4.85104 | 0.289851 | *Eftud1* |
| 10801024 | 9.75E-02 | -1.954847 | -5.575933 | 0.206982 | *Ankhd1* |
| 10739558 | 1.16E-01 | -1.831492 | -5.745995 | 0.190936 | *Slc16a5* |
| 10763149 | 1.29E-01 | -1.754662 | -5.850322 | 0.186735 | *Oas2* |
| 10781014 | 2.03E-01 | -1.424515 | -6.278827 | 0.180492 | *Pinx1* |
| 10752517 | 7.32E-01 | 0.358828 | -7.219147 | -0.038462 | *Ube2l3* |
| 10862262 | 9.48E-01 | -0.068269 | -7.290235 | 0.008122 | *Epha1* |
| 10723344 | 5.54E-02 | -2.361411 | -5.001924 | 0.282536 | *Saxo2* |
| Differentially expressed genes after Bonferroni correction (*P*<0.05/13) were in bold.  **^†^**Genes with the absolute of logFC more than 1. *SAXO2* is the alias for *FAM154B*. logFC=log2-fold change. | | | | | |

(B) IS-associated expression profile in the peripheral blood from GSE16561

| ID | *P*.Value | t | B | logFC | Gene |
| --- | --- | --- | --- | --- | --- |
| ILMN_2370336 | **2.73E-05** | -4.5014 | 2.276564 | 0.459546 | ***MS4A4A*** |
| ILMN_2371169 | **3.38E-04** | -3.776349 | -0.082001 | 0.524352 | ***ZYX*** |
| ILMN_1664265 | **1.22E-03** | 3.376374 | -1.263541 | -0.32408 | ***EPHA1*** |
| ILMN_1748090 | **2.55E-03** | -3.133166 | -1.945705 | 0.744073 | ***SLC2A11*** |
| ILMN_1755649 | 3.47E-02 | -2.154676 | -4.258781 | 0.257851 | *SLC16A5* |
| ILMN_1766560 | 4.15E-02 | -2.078398 | -4.408699 | 0.142878 | *ANKHD1-EIF4EBP3* |
| ILMN_1729167 | 5.31E-02 | 1.968328 | -4.616642 | -0.099689 | *EFTUD1* |
| ILMN_1677877 | 5.68E-02 | 1.937995 | -4.672181 | -0.148494 | *UBE2L3* |
| ILMN_2136133 | 7.40E-02 | -1.8149 | -4.889619 | 0.522348 | *PABPC1* |
| ILMN_2248970 | 2.04E-01 | -1.281603 | -5.678894 | 0.143708 | *OAS2* |
| ILMN_1812580 | 2.17E-01 | 1.245194 | -5.723429 | -0.071671 | *YDJC* |
| ILMN_1701248 | 5.37E-01 | -0.620598 | -6.28483 | 0.059425 | *TREM2* |
| ILMN_2182647 | 8.44E-01 | 0.196933 | -6.455927 | -0.013304 | *PINX1* |
| ILMN_2184884 | 8.80E-01 | 0.151501 | -6.471864 | -0.014215 | *SAXO2* |
| Differentially expressed genes after Bonferroni correction (*P*<0.05/14) were in bold. *SAXO2* is the alias for *FAM154B*. logFC=log2-fold change. | | | | | |

(C) IS-associated expression profile in the mouse spleen from GSE16561

| ID | *P*.Value | t | B | logFC | Gene |
| --- | --- | --- | --- | --- | --- |
| 10461587 | **1.57E-05** | -15.00848 | 3.87451 | 2.374638 | ***Ms4a4a*^†^** |
| 10438109 | **1.83E-03** | 5.777261 | -0.86837 | -0.710297 | ***Ube2l3*** |
| 10537770 | 8.08E-02 | -2.1555 | -4.98362 | 0.303017 | *Zyx* |
| 10554629 | 8.60E-02 | 2.107597 | -5.04777 | -0.298927 | *Eftud1* |
| 10565210 | 2.55E-01 | -1.276367 | -6.10478 | 0.14763 | *Saxo2* |
| 10433963 | 4.61E-01 | 0.794808 | -6.58888 | -0.175713 | *Ydjc* |
| 10428192 | 5.27E-01 | 0.676733 | -6.68161 | -0.062383 | *Pabpc1* |
| 10445781 | 7.10E-01 | 0.392096 | -6.85102 | -0.061075 | *Trem2* |
| 10544417 | 9.23E-01 | 0.101957 | -6.9342 | -0.015843 | *Epha1* |
| Differentially expressed genes after Bonferroni correction (*P*<0.05/9) were in bold. **^†^**Genes with the absolute of logFC more than 1. *SAXO2* is the alias for *FAM154B*. logFC=log2-fold change. | | | | | |
